# Supplementary material for: Climate adaptation by crop migration
Source: Nat Commun. 2020 Mar 6;11:1243. doi: 10.1038/s41467-020-15076-4 (PMC7060181; doi:10.1038/s41467-020-15076-4)
Supplement: Supplementary file 1 — Supplementary Information [file 41467_2020_15076_MOESM1_ESM.pdf]

Supplementary Information

Climate adaptation by crop migration

Sloat et al.

Includes:
Supplementary Figures 1-5
Supplementary Tables 1-7
Supplementary Note 1

Supplementary Figures

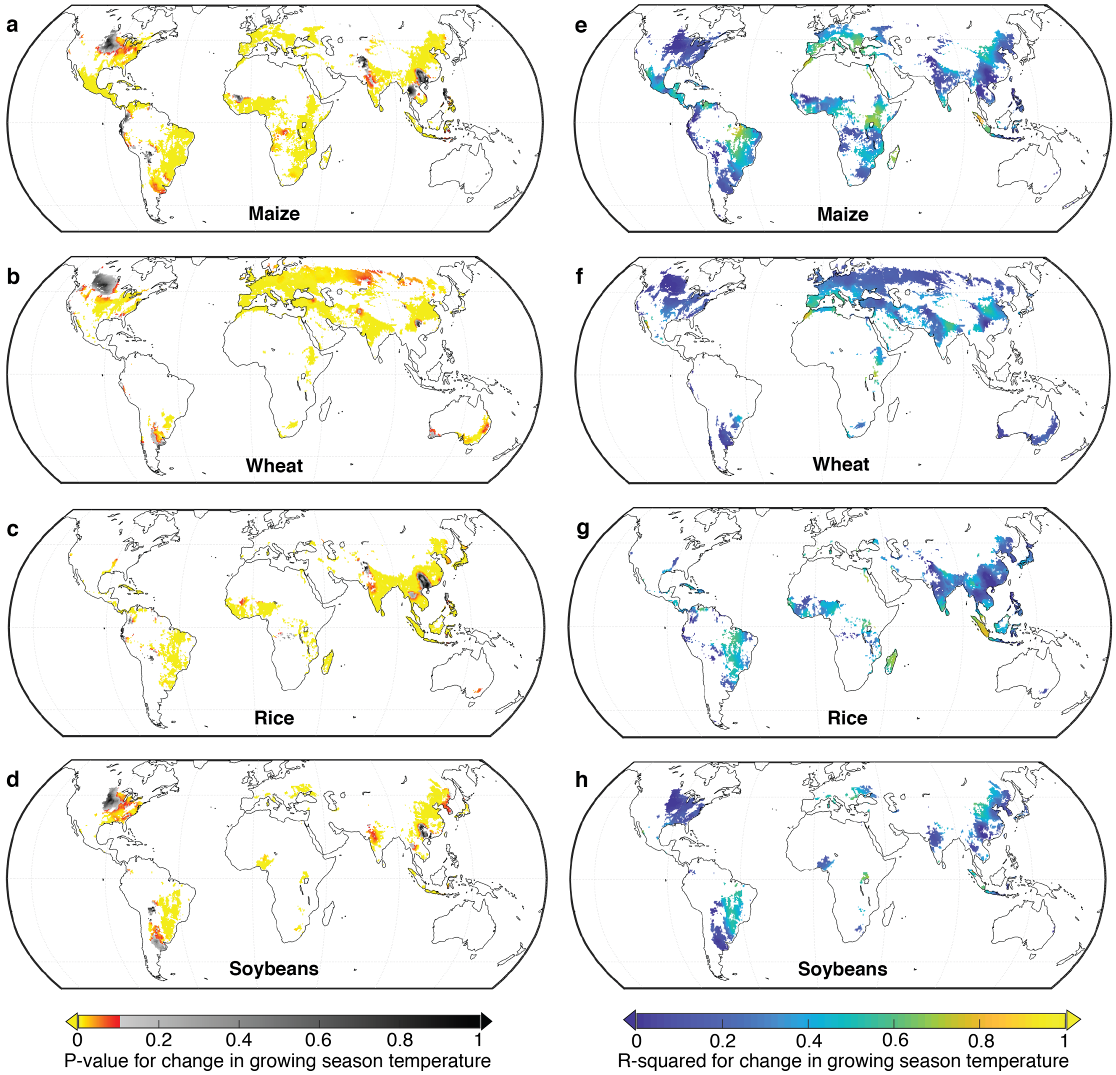

Supplementary Figure 1 | P-values (left) and R^2 values (right) for the linear regression of growing season average temperature changes from 1973-2012 (Figure 1 e – h) for maize, wheat, rice, and soybeans. Trends are calculated using linear regression, and all values are displayed across the top 98% of average harvested areas. Temperatures are averaged across multiple growing seasons (e.g. spring and winter wheat) where relevant.

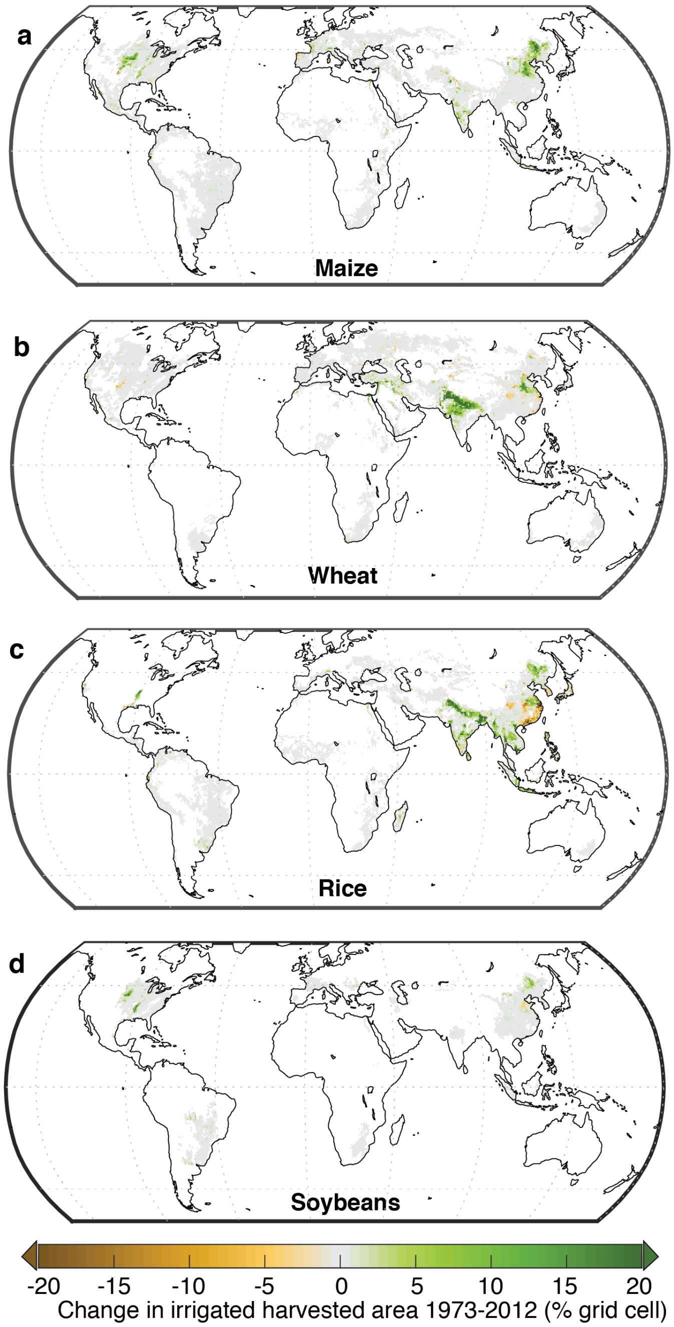

Supplementary Figure 2 | Trends in irrigated harvested area for maize (a), wheat (b), rice (c), and soybeans (d) between 1973–2012. Trends are calculated using linear regression, and all values are displayed across the top 98% of harvested areas. Locations in brown/orange are experiencing decreases in rainfed harvested areas, locations in green are experiencing increases, and locations in gray are experiencing near-zero trends.

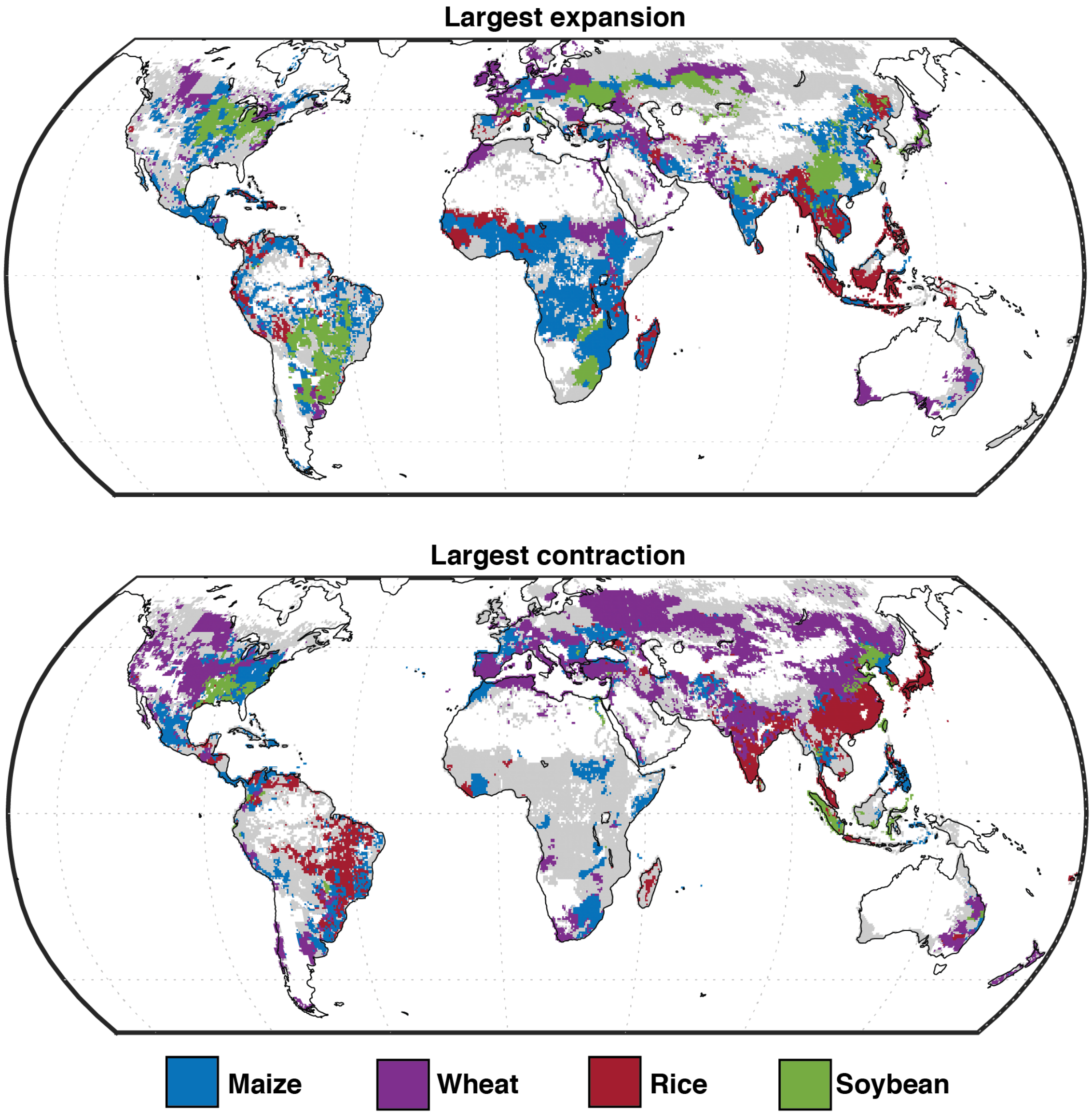

Supplementary Figure 3 | Global maps indicating which of the big four cereal crops gained the most (‘Largest expansion, top) or lost the most (‘Largest contraction, bottom) rainfed harvested area between 1973 and 2012. Changes in rainfed harvested area are found by linear regression and crop-specific rainfed harvested area change maps are shown in the main text Fig. 3. Maize is shown in blue, wheat in purple, rice in red and soybean in green. Areas that were analyzed but had changes in harvested area < 40 hectares are shown in gray.

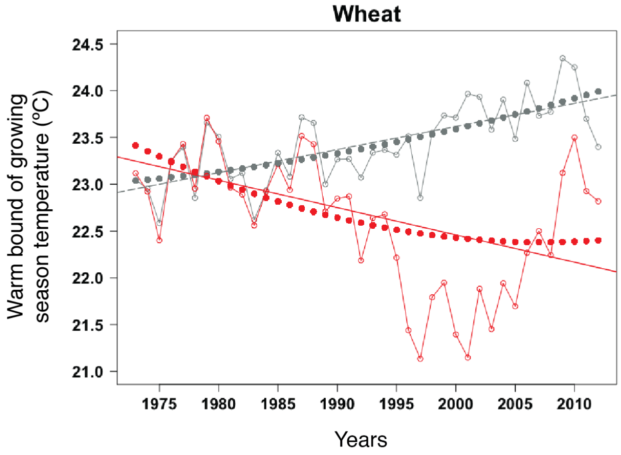

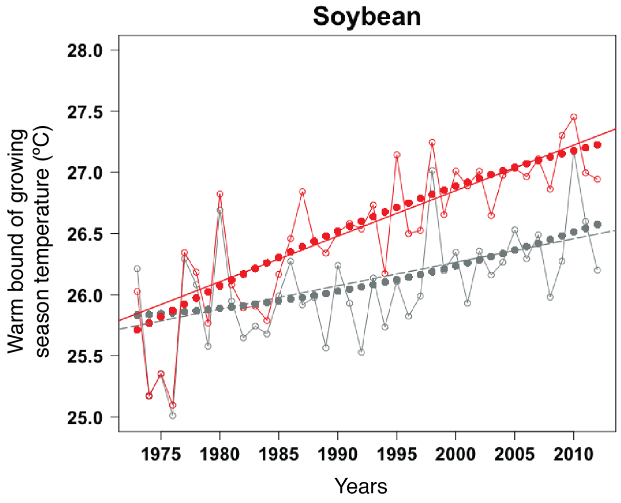

Supplementary Figure 4 | Quantile regression trends in the 95th percentile of growing season temperatures over time (1973–2012) for rainfed harvested areas fit with (dotted) and without (straight) a polynomial response term. These plots are identical to Fig. 4 in the main text but with the addition of the second-degree polynomial response term.

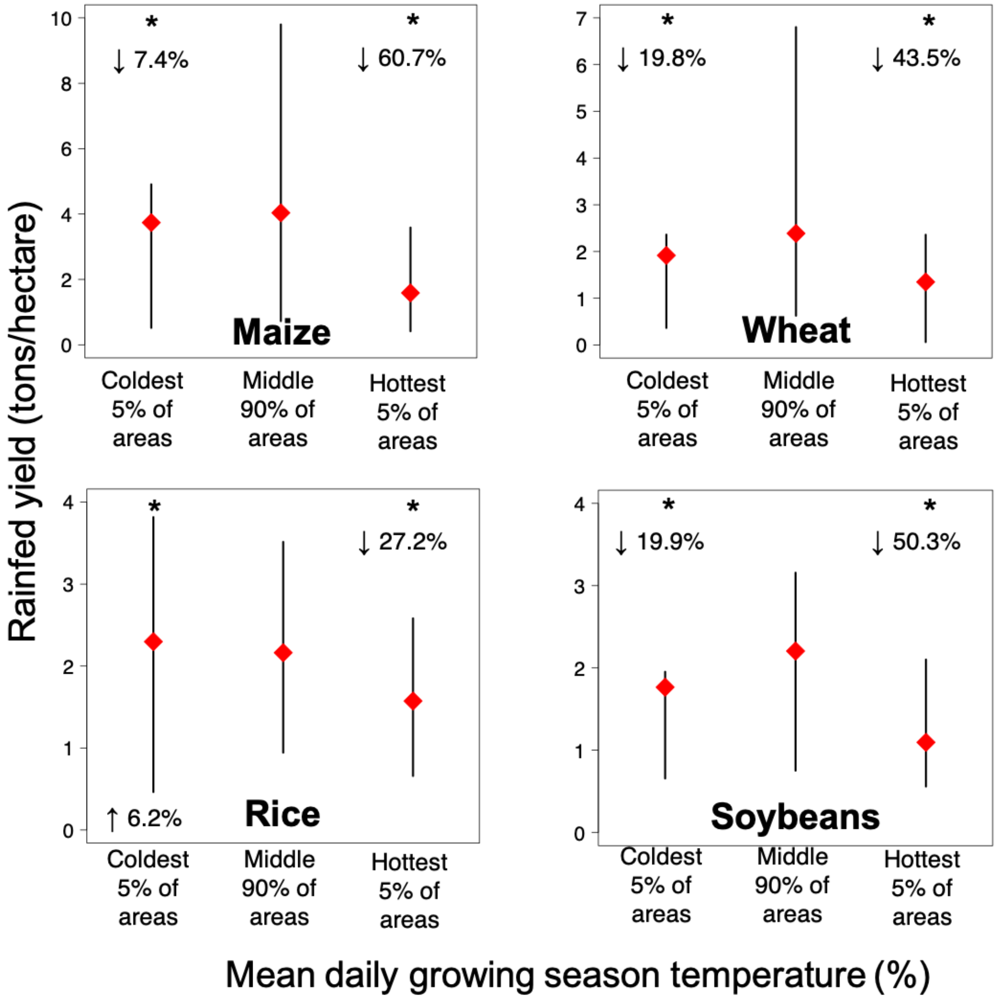

Supplementary Figure 5 | Rainfed crop yields and growing season temperatures. Rainfed yields and growing season temperatures are circa 2000 (1998 – 2002) for maize, wheat, rice, and soybean. The x-axis bins temperature into the coldest 5% (left), 5-95% (middle) and warmest 5% of growing season areas. Grid cells are weighted by crop areas, such that the red diamonds indicate the 50th percentile yield across all hectares and whiskers indicate the yields on the 5th and 95th percentile yield within each x-axis bin. Changes in the percent yield between the middle and outer x-axis bins are printed on the figure. Asterisks indicate a significant difference from the middle group (weighted means t-test $p < 0.05$). This plot shows that rainfed crop yields are lower in the hottest 5% of areas for all crops analyzed. Rainfed maize, wheat, and soybeans have lower yields in the coolest 5% of areas. Rainfed rice growing in the coolest 5% of areas has higher yields than rice growing in other areas.

Supplementary Tables

Supplementary Table 1 | Percentage harvested area experiencing warming and cooling trends. Based a linear regression of average growing season temperatures between 1973 – 2012 at significance levels of $P<0.1$.

	**Maize**	**Wheat**	**Rice**	**Soybean**
**Warming**	82.8%	99.8%	91.8%	68.2%
**Cooling**	0.0%	0.0%	0.0%	0.0%
**Not significant**	17.2%	0.18%	8.2%	31.8%

Supplementary Table 2 | Quantile regression model results for all crops at different percentiles (tau = 0.05, 0.9, 0.93, 0.95, 0.97, 0.99) for observed scenarios (weighted by dynamic harvested areas) and counterfactual scenarios (weighted by static, 1973-1977 harvested areas).

crop	tau	Coef	value	SE	t-value	p-value	value	SE	t-value	p-value
			OBSERVED				COUNTERFACTUAL			
maize	0.05	interc.	15.569	0.042	373.124	0.00000	15.596	0.043	365.136	0.00000
maize	0.05	slope	0.027	0.002	16.756	0.00000	0.030	0.002	18.107	0.00000
maize	0.9	interc.	26.774	0.025	1084.084	0.00000	26.699	0.029	925.927	0.00000
maize	0.9	slope	0.018	0.001	19.494	0.00000	0.018	0.001	15.375	0.00000
maize	0.93	interc.	27.331	0.029	935.579	0.00000	27.262	0.035	782.181	0.00000
maize	0.93	slope	0.013	0.001	11.563	0.00000	0.018	0.001	11.991	0.00000
maize	0.95	interc.	27.950	0.038	738.063	0.00000	27.842	0.043	645.009	0.00000
maize	0.95	slope	0.009	0.001	6.056	0.00000	0.017	0.002	9.601	0.00000
maize	0.97	interc.	28.608	0.044	651.099	0.00000	28.570	0.022	1287.678	0.00000
maize	0.97	slope	0.007	0.002	3.721	0.00020	0.016	0.001	21.783	0.00000
maize	0.99	interc.	30.816	0.046	667.165	0.00000	30.130	0.195	154.350	0.00000
maize	0.99	slope	-0.006	0.002	-2.562	0.01042	0.011	0.011	1.019	0.30840
wheat	0.05	interc.	0.451	0.033	13.626	0.00000	0.581	0.030	19.229	0.00000
wheat	0.05	slope	0.034	0.001	25.284	0.00000	0.028	0.001	22.057	0.00000
wheat	0.9	interc.	17.806	0.094	190.372	0.00000	17.883	0.122	146.876	0.00000
wheat	0.9	slope	-0.012	0.003	-3.868	0.00011	0.027	0.005	5.419	0.00000
wheat	0.93	interc.	21.978	0.163	134.877	0.00000	21.704	0.119	182.488	0.00000
wheat	0.93	slope	-0.080	0.006	-13.736	0.00000	0.028	0.005	5.353	0.00000
wheat	0.95	interc.	23.369	0.043	540.630	0.00000	22.937	0.041	561.949	0.00000
wheat	0.95	slope	-0.039	0.004	-10.578	0.00000	0.025	0.002	15.065	0.00000
wheat	0.97	interc.	23.916	0.037	655.004	0.00000	23.774	0.030	779.749	0.00000
wheat	0.97	slope	-0.002	0.002	-1.200	0.23027	0.025	0.001	19.081	0.00000
wheat	0.99	interc.	24.848	0.048	522.122	0.00000	24.827	0.026	939.283	0.00000
wheat	0.99	slope	0.016	0.002	6.966	0.00000	0.023	0.001	20.043	0.00000
rice	0.05	interc.	20.531	0.096	214.569	0.00000	20.435	0.088	231.112	0.00000
rice	0.05	slope	0.010	0.004	2.362	0.01816	0.022	0.004	6.177	0.00000
rice	0.9	interc.	27.769	0.021	1301.916	0.00000	27.773	0.022	1250.749	0.00000
rice	0.9	slope	0.015	0.001	18.672	0.00000	0.017	0.001	18.097	0.00000
rice	0.93	interc.	28.067	0.025	1130.064	0.00000	28.081	0.024	1147.968	0.00000
rice	0.93	slope	0.014	0.001	14.481	0.00000	0.016	0.001	16.813	0.00000
rice	0.95	interc.	28.358	0.030	948.401	0.00000	28.360	0.034	822.569	0.00000
rice	0.95	slope	0.012	0.001	9.724	0.00000	0.017	0.001	12.368	0.00000
rice	0.97	interc.	28.848	0.040	714.931	0.00000	28.810	0.039	747.128	0.00000
rice	0.97	slope	0.008	0.002	4.957	0.00000	0.018	0.002	9.869	0.00000
rice	0.99	interc.	29.730	0.088	339.628	0.00000	29.736	0.088	339.341	0.00000
rice	0.99	slope	0.012	0.004	3.427	0.00061	0.027	0.004	7.038	0.00000
soybean	0.05	interc.	17.251	0.063	275.589	0.00000	17.165	0.071	241.156	0.00000
soybean	0.05	slope	0.019	0.002	7.853	0.00000	0.024	0.003	8.094	0.00000
soybean	0.9	interc.	25.158	0.034	741.462	0.00000	25.151	0.040	634.283	0.00000
soybean	0.9	slope	0.043	0.001	35.031	0.00000	0.020	0.002	11.689	0.00000
soybean	0.93	interc.	25.571	0.035	723.761	0.00000	25.523	0.037	689.852	0.00000
soybean	0.93	slope	0.040	0.001	29.581	0.00000	0.020	0.002	12.582	0.00000
soybean	0.95	interc.	25.923	0.035	744.020	0.00000	25.835	0.040	650.929	0.00000
soybean	0.95	slope	0.037	0.001	28.145	0.00000	0.019	0.002	11.423	0.00000
soybean	0.97	interc.	26.372	0.045	590.926	0.00000	26.269	0.046	566.069	0.00000
soybean	0.97	slope	0.033	0.002	20.132	0.00000	0.019	0.002	9.336	0.00000
soybean	0.99	interc.	27.794	0.043	641.630	0.00000	27.054	0.211	127.961	0.00000
soybean	0.99	slope	0.017	0.002	7.163	0.00000	0.019	0.008	2.308	0.02099

Supplementary Table 3 | Average growing season temperature change (ºC) 1973 - 2012. Results are found by quantile regression for all crops at different percentiles (tau = 0.05,0.9, 0.93, 0.95, 0.97, and 0.99) for the observed model ('obs', dynamic rainfed harvested area weights) and the counterfactual model ('counter', static rainfed harvested area weights). P-values for the significant differences between the slopes of counter and obs models are included as well.

		**Change in temperature (ºC)**		**P-value**	
**crop**	**tau-value**	**obs**	**counter**	**counter < obs**	**obs < counter**
maize	0.05	1.07	1.21	1	0
maize	0.90	0.71	0.73	0.866	0.134
maize	0.93	0.54	0.70	1	0
maize	0.95	0.35	0.68	1	0
maize	0.97	0.27	0.65	1	0
maize	0.99	-0.24	0.43	0.84	0.16
wheat	0.05	1.36	1.13	0	1
wheat	0.90	-0.50	1.09	1	0
wheat	0.93	-3.22	1.11	1	0
wheat	0.95	-1.57	1.01	1	0
wheat	0.97	-0.09	0.99	1	0
wheat	0.99	0.64	0.93	1	0
rice	0.05	0.41	0.90	1	0
rice	0.90	0.60	0.67	1	0
rice	0.93	0.55	0.66	1	0
rice	0.95	0.46	0.67	1	0
rice	0.97	0.33	0.71	1	0
rice	0.99	0.49	1.09	0.866	0.114
soybean	0.05	0.75	0.97	1	0
soybean	0.90	1.72	0.80	0	1
soybean	0.93	1.60	0.79	0	1
soybean	0.95	1.48	0.77	0	1
soybean	0.97	1.32	0.78	0	1
soybean	0.99	0.68	0.78	0.316	0.684

Supplementary Table 4 | All 95th percentile quantile regressions in the manuscript were also done with a second-degree polynomial response term and the results are presented here.

crop	model	Coef	Value	Std. Error	t-value	P-val
maize	counterfactual	(Intercept)	27.83	0.0663	419.45	0.0000
		year	0.02	0.0072	2.61	0.0091
		$year^2$	0.00	0.0002	-0.25	0.8061
maize	observed	(Intercept)	27.90	0.0676	412.50	0.0000
		year	0.01	0.0066	1.25	0.2119
		$year^2$	0.00	0.0002	0.20	0.8419
rice	counterfactual	(Intercept)	28.36	0.0538	527.26	0.0000
		year	0.02	0.0057	2.84	0.0045
		$year^2$	0.00	0.0001	0.06	0.9538
rice	observed	(Intercept)	28.41	0.0458	620.97	0.0000
		year	0.00	0.0048	0.76	0.4461
		$year^2$	0.00	0.0001	1.74	0.0815
soybean	counterfactual	(Intercept)	25.93	0.0588	440.75	0.0000
		year	0.00	0.0065	0.73	0.4645
		$year^2$	0.00	0.0002	2.44	0.0148
soybean	observed	(Intercept)	25.78	0.0760	339.13	0.0000
		year	0.05	0.0073	7.30	0.0000
		$year^2$	0.00	0.0002	-2.24	0.0252
wheat	counterfactual	(Intercept)	23.04	0.0647	356.32	0.0000
		year	0.01	0.0071	1.48	0.1389
		$year^2$	0.00	0.0002	2.12	0.0344
wheat	observed	(Intercept)	23.50	0.0717	327.95	0.0000
		year	-0.06	0.0128	-4.78	0.0000
		$year^2$	0.00	0.0004	1.50	0.1338

Supplementary Table 5 | Rainfed harvested areas and growing season temperatures circa 1975 and 2010 for maize, wheat, rice, and soybean, summarized over regions of the world (continents and sub-continents).

	MAIZE				WHEAT				RICE				SOYBEAN			
	Rainfed harvested area (ha)		Growing season temperature (°C)		Rainfed harvested area (ha)		Growing season temperature (°C)		Rainfed harvested area (ha)		Growing season temperature(°C)		Rainfed harvested area (ha)		Growing season temperature (°C)	
**Region**	**1975**	**2010**	**1975**	**2010**	**1975**	**2010**	**1975**	**2010**	**1975**	**2010**	**1975**	**2010**	**1975**	**2010**	**1975**	**2010**
Oceania	66346	69553	22.04	22.34	8763213	13278721	15.20	15.67	51816	26248	21.34	22.08	66346	69553	22.04	22.34
Caribbean	230829	357358	24.38	24.91	0	0	NaN	NaN	259182	316833	24.88	25.48	230829	357358	24.38	24.91
Central America	7349830	6779607	20.90	21.87	492688	364470	19.04	20.11	302310	214899	24.72	25.50	7349830	6779607	20.90	21.87
Central Asia	162113	84147	20.33	21.07	12741985	12857333	5.08	5.96	212424	86486	17.54	18.47	162113	84147	20.33	21.07
Eastern Africa	7045327	13275161	23.47	24.31	864245	1826765	22.12	23.02	1048769	1931855	23.65	24.46	7045327	13275161	23.47	24.31
Eastern Asia	13417196	15472434	18.31	19.09	17172652	11370641	4.82	5.50	17045746	11056258	19.43	20.25	13417196	15472434	18.31	19.09
Eastern Europe	5501583	6311503	16.00	17.41	40971365	38533233	-0.79	0.19	202225	218380	17.38	19.12	5501583	6311503	16.00	17.41
Western Europe	1853252	1879675	14.98	16.07	6991723	9363339	8.30	8.99	3409	7284	14.20	15.74	1853252	1879675	14.98	16.07
Middle Africa	1808072	3666237	24.18	24.95	16488	12463	23.66	24.61	289500	612542	24.45	25.22	1808072	3666237	24.18	24.95
Northern Africa	731293	416857	25.42	26.97	5291031	5441269	21.45	22.82	222211	210274	25.83	27.36	731293	416857	25.42	26.97
Northern America	25385621	28338904	18.26	19.02	34438551	27508730	7.51	8.08	480133	212823	23.38	24.44	25385621	28338904	18.26	19.02
Northern Europe	0	3950	13.73	14.77	1989095	3386081	4.86	5.12	0	0	NaN	NaN	0	3950	13.73	14.77
South America	14667490	18318289	23.13	23.86	8193266	6678744	17.87	18.49	5574420	3363536	23.97	24.64	14667490	18318289	23.13	23.86
South-Eastern Asia	5630870	7357225	24.87	25.37	67642	53980	21.22	22.00	22555264	26985236	24.99	25.55	5630870	7357225	24.87	25.37
Southern Africa	4726267	2859407	19.99	21.19	1568657	494450	17.28	18.41	2362	1846	20.14	21.13	4726267	2859407	19.99	21.19
Southern Asia	5807580	7947007	25.58	26.28	18472591	14486176	18.81	19.74	34940760	28100307	24.66	25.37	5807580	7947007	25.58	26.28
Southern Europe	1087570	794398	17.27	18.99	6747298	3843391	10.90	11.91	134505	98202	17.05	18.69	1087570	794398	17.27	18.99
Western Africa	2132540	8249203	27.04	27.73	22472	74402	26.01	27.05	1704414	5116691	26.68	27.52	2132540	8249203	27.04	27.73
Western Asia	581187	564482	23.46	24.87	11245193	8845662	16.01	17.32	82995	91757	22.64	24.02	581187	564482	23.46	24.87

Supplementary Table 6 | Quantile regression model results for all crops at tau = 0.95 for ESRL data from 1979 - 2012 for observed and counterfactual scenarios.

crop	tau	Coef	value	SE	t-value	p-value	value	SE	t-value	p-value
			OBSERVED				COUNTERFACTUAL			
maize	0.95	interc.	28.04	0.03	808.10	0.00	28.02	0.04	758.33	0.00
maize	0.95	slope	0.01	0.00	7.96	0.00	0.02	0.00	11.11	0.00
wheat	0.95	interc.	22.89	0.05	471.17	0.00	22.78	0.04	546.16	0.00
wheat	0.95	slope	-0.02	0.00	-4.37	0.00	0.03	0.00	14.03	0.00
rice	0.95	interc.	28.52	0.03	955.63	0.00	28.55	0.03	938.26	0.00
rice	0.95	slope	0.01	0.00	10.02	0.00	0.02	0.00	11.44	0.00
soybean	0.95	interc.	26.14	0.04	585.03	0.00	26.05	0.04	624.38	0.00
soybean	0.95	slope	0.04	0.00	22.56	0.00	0.03	0.00	11.55	0.00

Supplementary Table 7 | Average growing season temperature change (ºC) 1979 - 2012. Results are found by quantile regression for all crops at tau = 0.95 for the observed model ('obs') and the counterfactual ('counter') models.

crop	tau-value	Change in temperature (ºC)		P-value	
		obs	counter	counter < obs	obs < counter
maize	0.95	0.56	0.90	1	0
wheat	0.95	-0.69	1.19	1	0
rice	0.95	0.59	0.72	1	0
soybean	0.95	1.72	1.02	0	1

Supplementary Note 1:

Changes in the cooler bound (5th percentile) are more complicated to interpret because an increase in growing season temperatures may make areas at this bound more favorable. Thus, experienced trends in the cool bound that are greater than the counterfactual suggest adaptation by migration, enhancing a beneficial change in climate by accelerating the movement towards more favorable intermediate temperatures. However, trends in the cool bound that increase more slowly than the counterfactual also suggest migration in response to warming, either by re-filling the crop's original thermal niche (if the slope remains ≥0) or an expansion of a crop's thermal niche into cooler conditions than where it was initially grown (if the slope is <0).

At the cool bound (5th percentile), the temperatures experienced by wheat are significantly greater than the counterfactual, again consistent with an adaptive migration of these crops into possibly relatively more preferable (in this case warmer) areas. Cool bound temperatures in the counterfactual scenarios for wheat increased by 1.13 ºC, while the experienced temperatures increased by 1.36 ºC (+0.23 ºC). Meanwhile, the cool bound temperature change experienced by maize, rice, and soybean (+1.07, +0.41, +0.75 ºC) was less than the counterfactual (+1.21, +0.90, +0.97 ºC), consistent with thermal niche re-filling. All quantile regression model results are presented in Supplementary Table 2. Temperature changes derived from quantile regression model slopes as well as P-values for the test of slope differences between counterfactual and experienced models are presented in Supplementary Table 3.
